# Exploring the complex pre-adaptations of invasive plants to anthropogenic disturbance: a call for integration of archaeobotanical approaches

**DOI:** 10.3389/fpls.2024.1307364

**Published:** 2024-03-15

**Authors:** Ginevra Bellini, Karin Schrieber, Wiebke Kirleis, Alexandra Erfmeier

**Affiliations:** ^1^ Department of Geobotany, Institute for Ecosystem Research, Kiel University, Kiel, Germany; ^2^ Cluster of Excellence ROOTS, Kiel University, Kiel, Germany; ^3^ Institute of Prehistoric and Protohistoric Archaeology, Kiel University, Kiel, Germany

**Keywords:** agropastoralism, anthropogenically induced adaptation to invade (AIAI), archaeobotany, evolution, invasive species, neolithic plant invasion hypothesis (NPIH)

## Abstract

Pre-adaptation to anthropogenic disturbance is broadly considered key for plant invasion success. Nevertheless, empirical evidence remains scarce and fragmentary, given the multifaceted nature of anthropogenic disturbance itself and the complexity of other evolutionary forces shaping the (epi)-genomes of recent native and invasive plant populations. Here, we review and critically revisit the existing theory and empirical evidence in the field of evolutionary ecology and highlight novel integrative research avenues that work at the interface with archaeology to solve open questions. The approaches suggested so far focus on contemporary plant populations, although their genomes have rapidly changed since their initial introduction in response to numerous selective and stochastic forces. We elaborate that a role of pre-adaptation to anthropogenic disturbance in plant invasion success should thus additionally be validated based on the analyses of archaeobotanical remains. Such materials, in the light of detailed knowledge on past human societies could highlight fine-scale differences in the type and timing of past disturbances. We propose a combination of archaeobotanical, ancient DNA and morphometric analyses of plant macro- and microremains to assess past community composition, and species’ functional traits to unravel the timing of adaptation processes, their drivers and their long-term consequences for invasive species. Although such methodologies have proven to be feasible for numerous crop plants, they have not been yet applied to wild invasive species, which opens a wide array of insights into their evolution.

## 1 Introduction

Since ancient times, human migration involved the intentional or unintentional transport of plant propagules, thereby significantly re-shaping the spatial distribution of the global flora (Hofman and Rick, 2018; Stephens et al., 2019). This process was initiated millennia ago with the introduction of species that with time became naturalised and thereby considered as part of the local floras in which they were introduced (Preston et al., 2004). These species were often associated with agriculture (Knörzer, 1971; Willerding, 1986; Whitehouse and Kirleis, 2014; Filipović et al., 2020; Dal Corso et al., 2022; Kirleis et al., 2022). During the last centuries, an ever-increasing global connectivity dramatically boosted the rate of plant species’ introductions (Seebens et al., 2017). Some of these rather recently introduced species have managed to successfully establish and reproduce in their non-native ranges, and spread rapidly within the landscape causing significant ecological and/or socioeconomic damage (Knapp et al., 2017). The underlying factors driving such species invasions have conventionally been explored through empirical studies primarily centered on contemporary populations and timeframes, with a few exceptions (Sheppard and Schurr, 2019; Brendel et al., 2021; Sheppard and Brendel, 2021). However, the conditions leading up to invasiveness often took place centuries, if not millennia earlier, and subsequently their traces have been attenuated by more recent shifts in the environment. In this review, we elaborate on integrative avenues of investigation at the crossroads of ecology and archaeology, which can draw us nearer to answering central queries regarding the origins of invasion success.

Since the 1960s, ecologists have been intrigued with understanding which factors contribute to plant invasion success, including species-specific traits and the environmental conditions shaping them (Baker, 1965). Researchers reported heritable divergence in germination characteristics (Donohue et al., 2010; Xia et al., 2011; Hock et al., 2015; Kreiner et al., 2022), growth and defense phenotypes (Schrieber et al., 2017; van Boheemen et al., 2019; Hock et al., 2019; Ollivier et al., 2020), reproductive capacities (Lachmuth et al., 2011; Turner et al., 2014; Helsen et al., 2020), and phenology (Wolfe et al., 2004; McGoey et al., 2020; Eckert et al., 2021) among invasive and native populations in numerous species, and provided evidence for corresponding shifts in their genomes (Lee, 2002; Lavergne and Molofsky, 2007; Wani et al., 2020; Sherpa and Després, 2021) or epi-genomes (Ainouche et al., 2004; Parisod et al., 2009; Mounger et al., 2021; Campoy et al., 2022). This variation arises from both stochastic and adaptive evolutionary processes, with research focusing more frequently on the latter (comprehensively reviewed in Kawecki, 2008; Verhoeven et al., 2010; Oduor et al., 2016; Bertelsmeier and Keller, 2018; Clements and Jones, 2021). Two mutually non-exclusive adaptive processes are considered to foster plant invasion success and are differentiated according to their spatio-temporal integration. Pre-adaptation refers to a process in which species traits that have evolved already in the native habitat also promote fitness in the invaded range, due to the similarity in environmental regimes, i.e., selective forces. In addition, plant species may undergo rapid post-introduction adaptation to evolve new traits in response to changes in selective regimes once they are exposed to the conditions of a novel habitat. Adaptations supporting plant invasions arise from various selective forces, particularly herbivory, competition, microbiota, climate and resource availability (discussed in depth in Erfmeier, 2013; van Kleunen et al., 2018; Sherpa and Després, 2021). Anthropogenic disturbance has the potential to alter all these environmental factors, and is thus considered as a key selective force in the evolution of invasive species, while pre-adaptation to anthropogenic disturbance has been proposed to generally foster invasion success (Hufbauer et al., 2011; Seastedt and Pyšek, 2011; MacDougall et al., 2018). However, the availability of empirical proof remains limited and fragmented, owing to the intricate character of anthropogenic disturbance in itself and the influence of additional evolutionary forces that shape the (epi)-genomes of contemporary native and invasive plant populations (see Chapter 4).

This review elaborates on the process of adaptation to anthropogenic disturbance (and in particular to agropastoral practices) that took place in the species’ native range with the establishment of agriculture several millennia ago, and later favoured their invasiveness upon co-introduction with such practices in novel habitats. Several frameworks have been proposed to explain how pre-adaptation can provide an advantage, once the species is brought to a new area (Hufbauer et al., 2011; MacDougall et al., 2018). We discuss in detail their assumptions, hypotheses, and proposed tests, and review the available empirical literature. We highlight that as the proposed favourable adaptations have partly taken place millennia ago with the onset of agropastoralism in the Neolithic, there is a need for expanding the temporal horizon of research beyond contemporary populations and the effects of recent environments on their genomes. Integrating theoretical frameworks with tangible archaeobotanical evidence may considerably deepen our understanding of past evolutionary transformations and their relationship with environmental drivers. Unearthing plant remains from ancient agropastoral contexts can help unveil past interactions between plant species and human activities, elucidating the past evolutionary processes that facilitated invasions, which are so far often unknown. This novel interdisciplinary approach may contribute to a more comprehensive understanding of the historical processes that shaped modern ecosystems, and simultaneously help developing strategies for managing present-day invasive species.

## 2 Anthropogenic disturbance, a central selective force in the adaptive evolution of plant invaders

### 2.1 The multiple facets of anthropogenic disturbance

Early studies in plant population ecology addressing effects of disturbance on plant performance focused exclusively on the individual scale and referred to disturbance as an event leading to the rapid and comprehensive destruction of biomass (Grime, 1979; Shea et al., 2004). As such, disturbance distinguishes clearly from the concept of a stressor, which is an environmental condition that has the potential to cause a reversible disruption of plant homeostasis (i.e., stress, Rout and Das, 2013). However, from an ecosystem-based perspective, disturbance comprises events that rapidly alter an ecosystem’s abiotic (e.g., resource availability, nutrient cycles) and biotic characteristics (e.g., species abundances and interactions) (Crisafulli et al., 2015). The impact of disturbance depends on the type (e.g., fire, storm, flood), intensity, spatial magnitude, timing, duration and frequency of the event (Shea et al., 2004; Miller et al., 2021). Natural disturbance events occur cyclically or seasonally as part of an environmental regime (Newman, 2019) while anthropogenic disturbance arises as a consequence of human activities such as subsistence activities, resource extraction or infrastructural development. In its effects, it often resembles natural disturbance, although amplified or modified: for example, livestock grazing could be seen as an intensified version of wild animal grazing (Walker, 2011). One significant difference between natural and anthropogenic disturbances is that while the former has, in most cases, a limited duration and a cyclical pattern, the latter can have long duration, very high and irregular frequency and wide spatial spread in/with cultural landscapes. Human alterations to landscapes impact biotic communities and biodiversity on a global scale. In Europe, the widespread deforestation that followed the introduction of agricultural practices during the Neolithic actually promoted an increase in species diversity, also thanks to the introduction of plants accidentally or deliberately associated with farming (Giesecke et al., 2019). However, nowadays land-use change is recognized as one of the major contributors to biodiversity decline (Millennium Ecosystem Assessment, 2005). Anthropogenic disturbances have the potential to alter ecosystem functioning and resilience in the long term, with legacy effects persisting even after centuries (Briggs et al., 2006; Battisti et al., 2016). Investigating past and recent adaptive responses of plant species and communities to disturbance is thus an actual and significant aim in evolutionary ecology research where invasive species serve as valuable model systems.

### 2.2 Direct effects of contemporary anthropogenic disturbance on plant invaders

Numerous empirical studies have shown that even moderate contemporary anthropogenic disturbance can favor the establishment, spread and competitive performance of invasive plant species. Much evidence has been gathered in the field with experimental (Hierro et al., 2006; Maron et al., 2013; Korell et al., 2017; Otfinowski and Coffey, 2022) and observational (Lake and Leishman, 2004; Oshima and Takahashi, 2020) approaches which targeted different types, intensities and frequencies of moderate disturbance, while focusing either on the plant community or single species level, and ranging in scale from 1 m^2^ up to > 1600 m^2^. Similar results have been obtained from mesocosm experiments (Kercher and Zedler, 2004; Corli et al., 2021), meta-analyses (Jauni et al., 2015) and studies considering both human development indices and invasive species abundance. For example, research showed that a region’s proportion of agricultural land, population density and per-capita Gross Domestic Product – all proxies for anthropogenic disturbance – positively correlate with its relative richness of naturalized and invasive plants (Essl et al., 2019). Disturbance is globally one of the key drivers of plant invasions. The often-observed positive relationship between anthropogenic disturbance and species invasion has been thoroughly investigated in the past decades and has led to the development of theories targeting the co-evolutionary history of plants with humans as cause for their invasion success.

### 2.3 Pre-adaptation to anthropogenic disturbance could favor plant invasions

There is consensus that past human activities such as mobility alongside the transport of goods and modification of the landscape can explain many of today’s species distribution patterns worldwide. By integrating large biogeographical datasets of introduced species (e.g., GloNAF - van Kleunen et al., 2019) with past and present socioeconomic indicators, researchers have recently begun to empirically uncover this complex interplay. A number of excellent studies demonstrated that species have a higher chance to naturalize somewhere if their native range has a long history of human occupation, and that the presence of anthropogenic disturbance in the past increases a region’s probability of successfully exporting these species (Monnet et al., 2020; Yang et al., 2021a). For example, numerous species have been brought from South-West Asia to the Mediterranean with the onset of agropastoralism (i.e., archaeophytes) (Zohary et al., 2012), as probably in the case of the invasive grass *Arundo donax* L., considered as one of the oldest invasive species (Hardion et al., 2014). Regarding more recent timeframes, a worldwide assessment of naturalized species revealed that areas occupied by the same European colonial power between the 16^th^ and 20^th^ centuries have floras more similar to one another than expected by chance, while regions geographically close but with a different colonization history can have quite distinct floras (Lenzner et al., 2022). This is due to the frequent trade between colonies and occupying country, but also due to the pronounced climatic, floristic and cultural differences among the interested European countries. In addition, species with a long cultivation history are much more likely to successfully naturalize and invade a new range (Kinlock et al., 2022). Although more recent human development (i.e., past 1900 CE) also re-shapes species distribution patterns (Pouteau et al., 2021), socioeconomic indicators from earlier time periods are better predictors in comparative studies (Essl et al., 2011). In summary, recent research evinced that the legacy of ancient human activities is still impactful on today’s ecological processes of species distribution, particularly invasions.

The mechanistic underpinnings for the association of plant invasion success with human (pre-)history involve i) the intentional or unintentional introduction of a sufficient number of propagules (Simberloff, 2009; Cassey et al., 2018; Faulkner et al., 2020), ii) the intentional cultivation of plant species (e.g., crops, forestry trees, ornamental plants – Guo et al., 2019; van Kleunen et al., 2020), and iii) intensive anthropogenic disturbance manifested in landscape transformations (e.g., through the establishment of pastures or crop fields, the re-direction of rivers, construction of barrages) and targeted eradication of native species (e.g., through weeding, deforestation or selective hunting) (Blumenthal et al., 2009; Ibáñez et al., 2021). The ability to tolerate specific anthropogenic disturbance is another fundamental prerequisite for an invasive species to thrive in its new range and it can emerge from post-introduction adaptation and/or pre-adaptation. Previous exposure and adaptation to anthropogenic disturbance during centuries of shared co-evolution between humans and introduced plant species is considered to be particularly important in this context (Hufbauer et al., 2011; Seastedt and Pyšek, 2011; MacDougall et al., 2018).

Plant species are generally adapted to the natural disturbance regime occurring in their native habitats. For example, tree species from the tropical savannah of the Brazilian Cerrado have developed thick barks and root sprouting as adaptation to the frequent fires characterizing the region (Simon and Pennington, 2012). Manmade disturbance, such as cultivation, can also lead to adaptation: during the process of domestication, for example, in ameliorated soils mycorrhizal symbiosis has become less essential for crop species than for their wild progenitors, as the former became adapted to the use of fertilizers (Martín-Robles et al., 2018). Likewise, populations of the wild plant *Solanum elaeagnifolium* Cav. which are periodically mown have evolved larger and faster germinating seeds than their unmown counterparts (Chavana et al., 2021). Species can experience nearly identical anthropogenic disturbance regimes in their native and invaded range, as many ecosystems and agropastoral habitats worldwide are altered by diverse but similar human actions (railroad building, application of cropping systems, forest management, grazing and mowing). Therefore plant species that are (epi)-genetically pre-adapted to human disturbance should be able to establish and thrive more successfully in novel anthropogenically-influenced habitats as compared to species that lack such pre-adaptations, a concept that has been summarized by Hufbauer et al. (2011) as “Anthropogenically Induced Adaptation to Invade” (AIAI). Other authors proposed that pre-adaptation to agricultural practices may explain the success of aggressive weeds like *Amaranthus tuberculatus* (Moq.) J.D. Sauer (Kreiner et al., 2022) and could be a driver for species invasions in crop fields on a global level (Ikegami et al., 2019). Finally, it has been suggested more specifically that invasive European species from agropastoral ecosystems succeed at invading other continents thanks to their introduction alongside anthropogenic “European style” disturbance regimes, to which they are pre-adapted (di Castri, 1989; La Sorte and Pyšek, 2009; MacDougall et al., 2018). The adaptation to this practice in Europe would have started in the Neolithic (~ 6000 BCE), when humans created semi-natural open habitats in previously forested areas (French et al., 2010). This “Neolithic Plant Invasion Hypothesis” (NPIH) could explain why many European species are problematic in agropastoral ecosystems all over the world. Some examples are *Cynoglossum officinale* L., and *Linaria vulgaris* Mill., which are invasive in the United States (Duncan and Williams, 2020; Blatt et al., 2022; Gaskin et al., 2022) and can be found in the archaeobotanical record, e.g., from well features in central Europe dating back to the Early Neolithic (Herbig et al., 2013). Independent of the exact native origin of invaders, gathering empirical evidence for a role of pre-adaptation to agropastoral practices in invasions has become an important goal in invasion ecology.

## 3 Existing approaches to test for the role of pre-adaptation to anthropogenic disturbance in invasions

Two complex methodological frameworks were proposed to empirically verify a role of pre-adaptation to anthropogenic disturbance in invasion success. Both present a comprehensive set of predictions and methods to test them based on native and invasive populations/plant communities. The methodological framework of Hufbauer et al (Hufbauer et al., 2011, AIAI) is the first to illustrate how previous exposure – and consequent adaptation – to anthropogenic disturbance might favor invasions of both animal and plant species without referring to a specific timeframe, while MacDougall et al (MacDougall et al., 2018, NPIH) selected a particular case of this scenario by focusing on adaptations that herbaceous plants might have developed in Europe with the onset of agropastoralism around 8000 years ago. **Table 1** summarizes the proposed predictions and tests while compiling empirical studies that have applied them.

**Table 1:** Overview of studies testing for pre-adaptation to agropastoral disturbance in European herbaceous species.

Approach	Prediction tested	Species	Support	Reference
AIAI	a) Populations of a given species are locally adapted to a specific regime of anthropogenic disturbance within their native range	*Centaurea stoebe*	+	Rosche et al., 2018
		*Euphorbia peplus*	+	Malíková et al., 2016
		*Amaranthus tuberculatus*	+	Kreiner et al., 2022
	b) A native population with (pre-) adaptation to a certain anthropogenic disturbance regime is identified as the genetic source for invasive populations	*Centaurea stoebe*	+	Marrs et al., 2008
	c) The invasive populations are subject to the same anthropogenic disturbance regime in the invaded range and thus maintained or even increased their adaptation to a given practice	*Centaurea stoebe*	NA	NA
NPIH	a) A major contribution of contemporary or post-introduction adaptation is ruled out	NA	NA	NA
	b) European species reach higher abundances in their invaded than native range under pastoral management	*Centaurea melitensis*	+/-	Moroney and Rundel, 2013
		*Centaurea solstitialis*	+/-	Hierro et al., 2017
			–	Hierro et al., 2013
			–	Xiao et al., 2016
	c) European species respond more positively to agropastoral management than native species	*Bromus tectorum, Cirsium arvense*	–	Connolly et al., 2017
		>10 species, see in original publication	–	Bellini et al., 2022
		>10 species, see in original publication	–	Broadbent et al., 2020
		>10 species, see in original publication	+/-	Maron et al., 2014
	d) European invasive species require agropastoral management for invasion	*Anthoxanthum odoratum, Cerastium fontanum, Pilosella officinarum, Holcus lanatus*	+	Jesson et al., 2000
		*Bromus tectorum, Carduus nutans, Hypericum perforatum, Poa bulbosa, Potentilla recta, Rumex acetosella*	+	Pearson et al., 2022
		*Anthriscus caucalis*	–	Wallace and Prather, 2016
		*Bromus tectorum, Cirsium arvense*	–	Connolly et al., 2017
	e) European invasive species are better colonizers of pastorally disturbed habitats than natives, regardless of propagule pressure	*Bromus tectorum, Cirsium arvense*	–	Connolly et al., 2017
	f) Agropastoral habitats outside of Europe are more invaded by European species than vice versa	NA	NA	NA

The category “approach” describes to which paper (either Hufbauer et al. (Hufbauer et al., 2011 - AIAI) or MacDougall et al. (MacDougall et al., 2018 - NPIH)) first describes the prediction that is tested in the referenced paper. For each prediction we report the studies we could find, highlighting the studies species and whether the result supported (+) or contrasted (-) the prediction (NA= data not available). This table includes all studies that are listed on the Web of Science platform given the following search criteria: plant* AND (invasi* OR alien OR non-native) AND (pre-adaptation OR preadaptation OR adaptation) AND (disturb* OR pastoral* OR agro*).

### 3.1 Are native populations with local pre-adaptations to anthropogenic disturbance the sources for invasion?

To test whether pre-adaptation to anthropogenic disturbance contributes to invasion success, Hufbauer et al. (2011) suggest a multi-step approach taking into account intra-specific differentiation in disturbance responses of a species in both its native and invaded range. It consists of **a)** gathering evidence that populations of a given species are locally adapted to a specific regime of anthropogenic disturbance within their native range, **b)** identifying a native population with a proper (pre-)adaptation to a certain anthropogenic disturbance regime as the genetic source for invasion and **c)** showing that the corresponding invasive populations are subject to the same anthropogenic disturbance regime in the invaded range and thus maintained or even increased their adaptation to a given practice. Given the complexity of the required experiments and the depth of phenotypic and genetic analyses required to test these predictions, empirical evidence for plants is mostly limited to one or two of these predictions – on which we will elaborate in the next section – while to our best knowledge compiling support has been gathered for no species.

The species that provide some evidence for two or more predictions of the AIAI hypothesis belong to different families and life forms. For example, the herbaceous plant *Euphorbia peplus* L. can be found in its native range both within habitats where the vegetation is harvested once a year and in undisturbed habitats. An empirical test showed that the populations originating from the former habitats have higher compensatory growth capacity than populations from the latter (Malíková et al., 2016). In Australia, where the species is invasive, it is mostly found in gardens and other disturbed habitats (Orchard, 1994). Likewise, *Amaranthus tuberculatus* can inhabit either agricultural or semi-natural ecosystems in its native range North America, with individuals from the former habitats outperforming those from the latter in multiple traits linked to reproduction. More recently, the species started expanding aggressively within its native range exclusively as an agricultural weed (i.e., Waselkov and Olsen, 2014), which indicates that populations pre-adapted to agricultural disturbance are capable of rapidly proliferating in novel habitats with matching disturbance regimes (Waselkov et al., 2020; Kreiner et al., 2022). For the Asteraceae *Centaurea stoebe* L., there is detailed information available regarding population differentiation across native habitat types, genetic sources of invasion and the response of invasive populations to disturbance. The native range of Eurasia harbors both diploid, preferably inhabiting semi-natural habitats, and tetraploid populations, which are more commonly found in human-altered habitats (Otisková et al., 2014). In the invaded range of North America, the plant is present only with tetraploid populations (Mráz et al., 2011; Rosche et al., 2016), probably originating from Romania, Bulgaria and Slovenia (Marrs et al., 2008). In an experiment investigating the effects of disturbance on populations from both ranges and ploidy levels (American tetraploid vs. European tetraploid vs. European diploids), tetraploids from both ranges showed a higher resprouting success after clipping than diploids, indicating that pre-adaptation to disturbance may be one component that contributed to invasion success of the species (Rosche et al., 2018).

### 3.2 Are invaders originating from Europe pre-adapted to agropastoral disturbance?

In the specific case of European herbaceous plants, MacDougall et al. (2018) propose six predictions to be tested in combination for verifying that pre-adaptation to agropastoral disturbance is indeed central for invasion success (**Table 1**). Their global approach aims at demonstrating that:

**a)** European invaders from native and invasive populations perform similarly under disturbance, i.e., a major contribution of post-introduction adaptation can be excluded.
**b)** European invaders reach higher abundances in their invaded rather than native range under agro-pastoral management.
**c)** European invaders respond more positively to agropastoral management than native species.
**d)** European invaders require agropastoral management for invasion.
**e)** European invaders are better colonizers of disturbed agropastoral habitats than natives, regardless of propagule pressure.
**f)** agropastoral habitats outside of Europe are more invaded by European species than vice versa.

Until today, most of the evidence gathered from empirical studies provides little support or even contrasts predictions of the NPIH, although never explicitly aiming at testing it, and to our best knowledge no compiling evidence is available.

Since the scientific community nowadays agrees that post-introduction adaptation is ubiquitous (Reznick et al., 2019), it is nearly impossible to empirically rule out that this force plays a significant role in an invasion process, i.e., it is unfeasible to find evidence for **prediction a)**. **Prediction b)** has been addressed with both experimental and observational studies, specifically in the genus *Centaurea*, yielding varying results. For *Centaurea solstitialis* L., a germination experiment reported no difference in density between native and invaded ranges under disturbance (Hierro et al., 2013). A later study with mature plants found a difference in disturbance-promoted density between ranges, but the higher density was observed in the native range (Hierro et al., 2017). For *Centaurea stoebe*, a field experiment with small-scale disturbance found no higher density in disturbed plots in either the native or invaded range (Maron et al., 2013). Finally, *Centaurea melitensis* L. occurs in higher density in some regions of its invaded range, but its abundance correlated negatively with the disturbance score of each site (Moroney and Rundel, 2013). **Prediction c)** has been tested with several large-scale multi-species experiments, which provided mixed support. Researchers studied the response of European invaders to different disturbance regimes such as biomass removal, nutrient addition and soil disruption, and they found no (Connolly et al., 2017; Broadbent et al., 2020; Bellini et al., 2022) or only partial (Maron et al., 2014) support for this prediction. With regard to **prediction d)**, two multispecies experiments that focused on plant responses to soil disruption indeed found that some European species were unable to establish in undisturbed soil (Jesson et al., 2000; Pearson et al., 2022). In contrast, a single-species experiment in New Zealand found evidence against this prediction, with higher abundances of the invasive European plant *Anthriscus caucalis* M. Bieb. in undisturbed than in disturbed plots (Wallace and Prather, 2016). This result is similar to the seed-addition experiment of Connolly et al. (2017), who showed that two European invasive species were able to establish in plots without disturbance and did not benefit more from disturbance than native species, even with a standardized amount of added seeds (**predictions d) and e)**). There are some studies that complement field experiments with other approaches, such as empirical and individual-based modelling. Combining results from three experiments, Xiao et al. (2016) found that the establishment of *Centaurea solstitialis* under competition depended on the interaction between disturbance and origin of the competitors, therefore providing mixed support for **prediction d)**. The species failed at establishing in absence of disturbance but was able to establish under strong disturbance only when competing with American grasses. For **prediction f)**, we still lack biogeographical studies that analyze global invasion patterns by habitat type with a particular focus on European species and agroecosystems. Gathering further empirical evidence to support these predictions and the associated assumptions is fundamental to uncover the role of past anthropogenic disturbance on invasive species traits and genomes.

## 4 Conceptual and practical challenges in evaluating whether successful invaders were pre-adapted to anthropogenic disturbance

While there is support for a role of pre-adaptation to anthropogenic disturbance in invasion success in the recent past (i.e., for the AIAI hypothesis), evidence for the more ancient time frames postulated by the NPIH is lacking. Europe is the native range of a vast number of species that naturalized elsewhere, ranking second after Asia in terms of absolute numbers of species, but with a much higher observed number of naturalizations than expected (288% higher for Europe, 52% higher for Asia) (van Kleunen et al., 2015). Given that the importance of historical association with humans for the invasion success of these species is evident (Monnet et al., 2020; Yang et al., 2021a) and likely causes feedback on recent species traits and invasion processes (Crosby, 1986; di Castri, 1989; MacDougall et al., 2018), further research into the AIAI and NPIH using methods and perspectives derived from interdisciplinary approaches is urgently required. In the following sections, we will outline i) why empirical research might benefit from focusing on ancient plant populations in addition to contemporary ones, and ii) how refining the underlying theory based on progress in archaeological research may help to develop more differentiated predictions to be tested. We finally highlight that integrating archaeology with ecology offers excellent opportunities to study past genetic and morphologic changes over large timeframes and possibly test for their association with anthropogenic environmental transformations.

### 4.1 Scratching the surface: on the complexity of evolutionary processes shaping species traits in contemporary populations

Although the events that lead to the pre-adaptation of plant species to anthropogenic disturbance in sense of the AIAI and NPIH mainly happened thousands of years ago, the suggested and implemented experimental approaches towards this issue focus exclusively on trait comparisons in contemporary plant populations (see Section 3.1). However, the phenotypic traits of these populations result from a plethora of mutually non-exclusive adaptive and stochastic (e.g., random sampling of genetic lineages, founder effects, allele surfing, population admixture) evolutionary processes, the relative importance of which varies across both a temporal and spatial scale (Keller and Taylor, 2008; Schrieber and Lachmuth, 2017; Liao et al., 2020; Sherpa and Després, 2021; Wilde et al., 2021; Kreiner et al., 2022). All these selective and neutral processes shape species traits and the underlying (epi)-genetics from the moment of introduction right until the present. As such, post-introduction evolutionary processes will blur pre-adaptation effects and substantially contribute to the divergence in traits we observe in recent populations. Post-introduction evolution cannot be ruled out in any experiment dealing with recent populations, specifically not for rather short-lived grassland species originating from Europe that established in their invaded ranges starting from 1500 CE. This does also apply to the resident native communities, which are in the focus of predictions of the NPIH as well. Given that adaptive change can happen within only a few years, also native plant communities should have continuously adapted to agropastoral disturbances or any other selective agent. Finally, evolutionary processes foster divergence in species traits not only between, but also within their native and invasive distribution range. Such divergence was observed among populations separated only by a few kilometers (Adhikari et al., 2021), across latitudinal clines (Liu et al., 2020; Yang et al., 2021b) or for invasive populations among leading edges and origins of initial introduction (Chuang and Peterson, 2016), and this variation is often not accounted for in the studies addressing the NPIH. In summary, purely ecological experiments cannot explicitly test the NPIH, since they cannot attribute trait divergence to a specific evolutionary force (adaptive or neural), selective agent or – most importantly – to a concrete timeframe.

### 4.2 Revisiting the Eurocentric view on agropastoral development

An important assumption underlying specifically the NPIH is that non-European plant communities were naïve to the “European style” agropastoral disturbance co-introduced by the European settlers alongside some of today’s most problematic plant invaders. This assumption is questionable, since natural disturbances partially resemble the selection pressures exerted by agropastoral disturbance (see Chapter 3) and may thus lead to similar adaptations of native plant communities in invaded continents (Mercuri et al., 2018).

**Figure 1:**
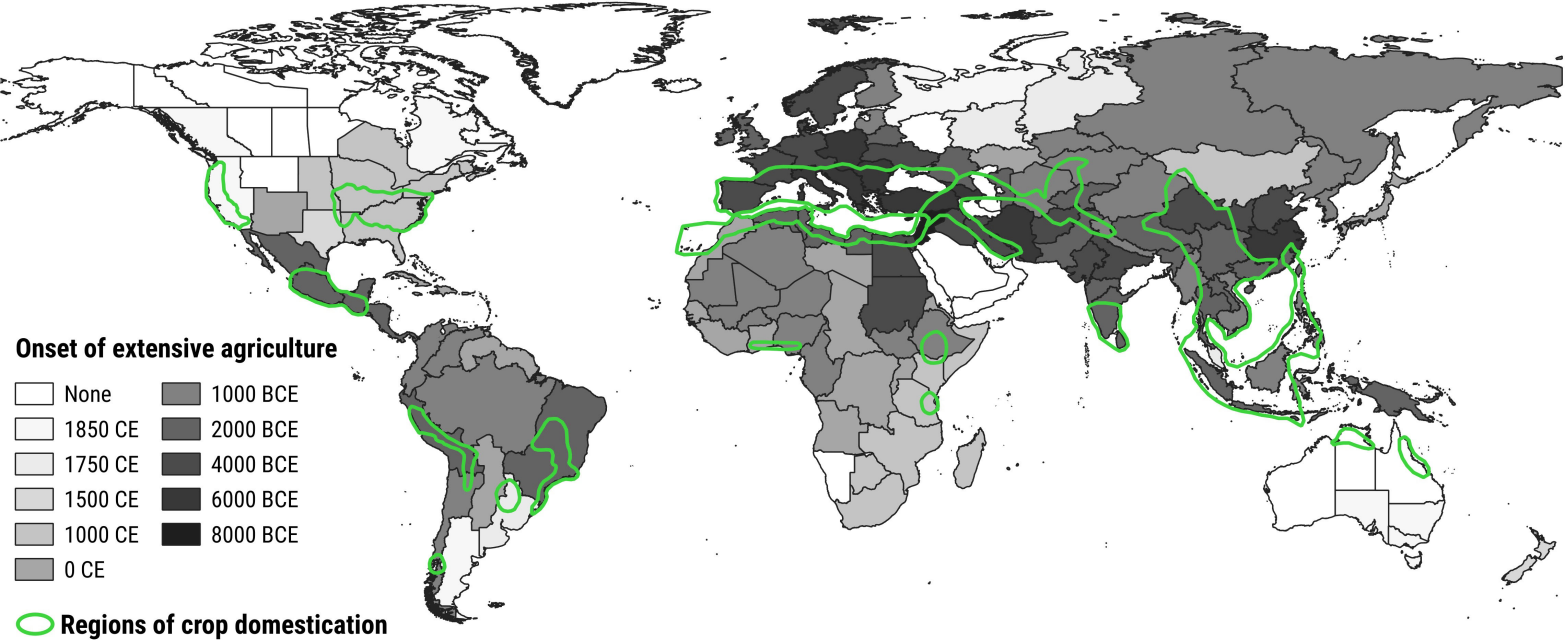
Development of extensive agriculture through time, and regions of crop domestication. The onset of extensive agriculture is represented by the color of the region, with darker shades indicating earlier onsets (Stephens et al., 2019). Authors defined onset as the earliest time point in which the practice was common in 1-20% of the region. Regions of crop domestication are outlined in green (Maxted and Vincent, 2021).

Second, it is reasonable to ask whether these native communities indeed never experienced agropastoral disturbance. Although European agriculture and pastoralism have a millennia long history characterized by technological innovations, species domestication and landscape change, they are definitely not an *unicum* in global history (Purugganan and Fuller, 2009). In fact, agriculture and pastoralism surged independently during pre-history (period of time before written records) in many different areas worldwide (Stephens et al., 2019) and nowadays there are twelve areas (subdivided in smaller sections) which are recognized as crop domestication regions and thus strictly associated with agronomical management (Smith, 2006; Purugganan and Fuller, 2009; Neves and Heckenberger, 2019; Maxted and Vincent, 2021) (**Figure 1**). Convergent evolution in plant adaptations has been proven in several contexts (Keeley and Pausas, 2018; Artur and Kajala, 2021) and it is thereby reasonable to assume that many of the species exposed to agropastoral disturbance worldwide eventually adapted to crop plant cultivation and livestock rearing as well (Bellini et al., 2022). There is indeed broad evidence that European colonizers found already human-altered open ecosystems characterized by species that likewise had adapted to agropastoral disturbance. This circumstance is often ignored when considering global plant invasions from a European perspective. In the South American Andes, for example, the first traces of domesticated plants and agricultural practices date back to 8000 BCE (Piperno and Pearsall, 1998; Nascimento et al., 2020), with some cultures implementing comprehensive landscape modifications such as terracing (Denevan, 2001) and forest clearing (Sarmiento, 2002). Many crop species with nowadays global relevance were domesticated in the region, such as potato (*Solanum tuberosum* L.), manioc (*Manihot esculenta* Crantz), and sweet potato (*Ipomoea batatas* (L.) Lam.) around 8000-6000 BCE, followed by quinoa (*Chenopodium quinoa* Willd.) and some varieties of squashes (*Cucurbita* spp.) around 5800-4400 BCE (Pearsall, 2008). In addition, native mammal herbivores such as camelids were domesticated since 4000-3500 BCE and used as source of sustenance and textile fibers (Flores Ochoa et al., 1994; Yacobaccio and Vilá, 2016). The presence of agriculture and pastoralism impacted the local ecosystems’ characteristics through biomass removal, soil disruption and manuring, in a similar manner to other agroecosystems worldwide. As a logical consequence, South America is also the native range of numerous aggressive plant invaders, such as *Galinsoga parviflor*a Cav., *Parthenium hysterophorus* L., and *Axonopus fissifolius* (Raddi) Kuhlm which are present in agropastoral ecosystems around the world (Warwick and Sweet, 1983; Parker, 2012; Tsiamis et al., 2017). In North America, the modification of the landscape by native human populations began already around 6000 BCE (Nelson et al., 2006; Smith and Yarnell, 2009) with the creation of large open environments to provide grazing ground for the large herbivores they hunted, such as bison (*Bison* spp.), elk (*Cervus* spp.) and deer (*Odocoileus* spp.). In the eastern United States, we find evidence of cultivation already 5000 years ago (Mueller, 2017), with the domestication of sumpweed (*Iva annua* L.) (McLauchlan, 2003), maygrass (*Phalaris caroliniana* Walter) and erect knotweed (*Polygonum erectum* L.) (Mueller, 2017). With the spread in agricultural practices, indigenous people started managing cultivation areas with soil tilling, mulching, removal of rocks, and creation of field boundaries (Deur, 2005). Over time, this frequent environmental management promoted the evolution of tolerance traits in some species, such as *Chenopodium* spp., *Helianthus annuus* L. and *Carya* spp (McLauchlan, 2003; Black et al., 2006; Johnson and Abrams, 2017), many of which are successful invaders in Eurasia. Furthermore, it should be noticed that some practices applied in Europe were common to other regions worldwide and changed over time (Huisman and Raemaekers, 2014; Filatova et al., 2021; Kirleis, 2022). One prime example is the creation of agricultural terraces, which allow the exploitation of hillsides and can be found in the Mediterranean (e.g., North-Western Italy, Southern Greece, starting ~2000 years ago), the Peruvian Andes (starting ~4000 years ago), and Hawai’i (starting ~1500 years ago), among others (Sereni, 1961; Allen, 1991; Denevan, 2001; Brandolini and Cremaschi, 2018; Brown et al., 2020). There are of course some important differences as well, such as the usage of planting sticks instead of ploughs by Native North Americans (Morrison et al., 1996), a technique that suited well the crops they planted (e.g., tubers and maize) and aimed at maintaining soil fertility while still causing soil disruption (Mt. Pleasant, 2015).

Finally, (pre-)historical European agropastoral practices cannot be considered as uniform, since distinct practices of the early European settlers from different geographic regions had a profound influence on the type of agropastoral practices and domesticated crops and animals in the colonized areas (Saloutos, 1976; Cannon, 1991). For example, in North America during the 1800s immigrants from Norway grew rye, barley, turnips and potatoes, while settlers from Germany focused on wheat, orchards, vineyards, and those from Switzerland invested in cattle rearing and cheese making (Saloutos, 1976). These cultural differences eventually translated into distinct types, intensities and timings of disturbance in the colonized areas, which produced profoundly different effects on local vegetation, as experiments on contemporary effect of disturbance of plant performance have shown (see Section 2.2). Both these factors taken together hamper a standard definition for the “European-style” agropastoralism highlighted in MacDougall et al. (2018). In summary, despite some differences between macro-regions, the global variability of practices at a regional scale does not allow us to classify a species as pre-adapted to agropastoralism just based on its continent of provenance.

### 4.3 Addressing open questions from an historical perspective

Future studies addressing the AIAI and NPIH should aim at disentangling ancient adaptations to anthropogenic disturbance from more recent adaptive or stochastic evolutionary change, while accounting for the cultural diversity of agropastoral practices that created such adaptations. To this end, we need to gather evidence on whether adaptations did indeed happen in a specific historical period, in response to which particular disturbance regimes and at which spatial scale (e.g., local, regional, continental). These issues cannot be comprehensively tackled with experiments on contemporary plant populations and thus require additional research on remains of populations dating back to centuries or even millennia before present. In combination, experiments on recent populations and research on ancient material will provide a more holistic view on the role of pre-adaptation in plant invasion success.

In this sense, a promising yet so far hardly employed resource in the contexts of the AIAI and NPIH frameworks is represented by the plant material that is recovered in archaeological excavations, although its potential was identified some time ago (Willerding, 1978; van Zeist et al., 1991). We believe that archaeobotany, the study of ancient plant remains, can make a valuable contribution to the field of invasion ecology, particularly in relation to hypotheses that focus on plant mobility in the past. Next to the movement of crops (e.g., Kirleis and Fischer, 2014; Filipović et al., 2020; Dal Corso et al., 2022; Kirleis et al., 2022), the accompanying weed species are of core interest in archaeobotanical research since their presence can provide supplementary information on ancient connectivity, past agropastoral practices and disturbance of natural vegetation (Knörzer, 1971; Willerding, 1983; Kreuz, 1994; Knörzer, 1998; Rösch, 1998; Bogaard et al., 1999; van der Veen, 2005). The integration of ancient DNA analyses for reconstructing past genomes currently focuses on crop domestication (Brown et al., 2009; Czajkowska et al., 2020; Filatova et al., 2021), but could be efficiently applied to wild species as well. The incorporation of ecological and archaeobotanical knowledge in a multidisciplinary approach would allow us to answer many emergent questions about adaptation processes in invasive species and the limits and conditions under which they have developed.

## 5 Future research routes: combining materials, methods and knowledge from archaeobotany and plant ecology

**Figure 2:**
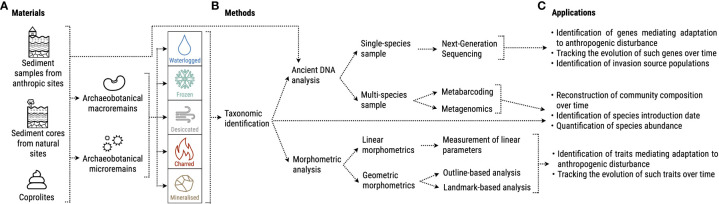
Overview of possible **(A)** materials, **(B)** methods and **(C)** applications of archaeobotanical knowledge to the study of invasive plants’ past evolution.

In the following sections, we will compile research avenues at the interface of archaeobotany and plant ecology by illustrating sample materials, analytical methods, and concrete applications to investigate how local development of agropastoral practices affected species genomes and traits (**Figure 2**).

### 5.1 Archaeobotanical remains

Archaeobotanical remains can be divided into two categories: macroremains such as seeds, fruits, wood pieces, storage tissues (roots, bulbs), and microremains such as pollen, starches, and phytoliths (Weiss and Kislev, 2007; Fuller and Lucas, 2014) (**Figure 2A**). A series of processes contributes to their preservation from decomposition, namely: i) waterlogging; ii) freezing; iii) desiccation; iv) charring; v) mineralization (Fuller and Lucas, 2014) (**Figure 2A**). Remains can be found within excavated sediment or other media such as fossilized dung (Linseele et al., 2013; Jakobitsch et al., 2023) (**Figure 2A**). Most archaeobotanical investigations are performed on past human settlements, which can lead to a bias in the species that are found, favoring high proportions of crops or useful species and rather small amounts of wild species without service to human beings (Jones, 1985; Fuller and Lucas, 2014). Analyzing sediment layers from archaeological and geological trenches at different depths rather allows the reconstruction of plant usage and vegetation changes over time (e.g., Kroll, 1983; Mercuri, 2008). Sediment sequences extracted from more natural environments, such as lake sediments, can provide samples with high temporal resolution that represent the taxonomic composition of the past local vegetation and its change over time (Wieckowska et al., 2012; Sadori, 2013; Feeser et al., 2016; Parducci et al., 2017; Wagner et al., 2023).

### 5.2 Analytical methods and ecological applications

Once retrieved, remains such as seeds must be first taxonomically identified and quantified by comparing them with a reference collection, i.e., an assortment of modern plant parts collected from known species with the help of instruments such as microscopes and stereoscopes (**Figure 2B**). Depending on the type of remain, identification to the species level is not always possible. In the case of phytoliths, for example, many studies have been carried out on cultivated species (Ball et al., 2016) although more work recently has focused on wild species (Le Moyne et al., 2023). The taxonomic identification of archaeobotanical samples could provide comprehensive information about species presence/absence, relative abundance, and even the introduction date in the case of non-native species. Several archeological studies have already provided valuable records of introduced and invasive plants in past contexts. For example, the arrival of non-native herbal species happened alongside the introduction of agriculture in Europe by the first Neolithic farmers (Kreuz, 1994, 2012). The analysis of a comprehensive assemblage of plant macroremains from Czech Republic revealed several introduction waves from the Neolithic until the early Middle Ages, with some of the introduced species being considered nowadays invasive (e.g., *Atriplex sagittata*, *Digitaria ischaemum* and *Echinochloa crus-galli*) (Pokorná et al., 2018). Another study on macro-remains, pollen and historical records from eastern France identified two periods of frequent introductions of non-native and invasive species: from the end of the Neolithic until the late Bronze Age, with arable weeds brought from the Mediterranean area, and a second after 1500 CE with ruderal species coming from outside the continent (Brun, 2011). Identification of plant remains can provide information on past human presence and management practices as well. For this purpose, the study of pollen in sediment is particularly suitable (Mercuri, 2014). Pollen analyses of two 2000-year-old sediment records from an archaeological site in the United States, show a high presence of ancient crops and indicate that the area was largely deforested (McLauchlan, 2003). A similar conclusion was reached by the analysis of Bronze-age pollen remains from northern Italy, which highlighted a thinning of forested areas due to the establishment of human settlements (Mercuri et al., 2015). Studies on pollen cores can also correlate human population demographics and activities with variation in plant species composition, identifying periods characterized by e.g., cultivation expansion, abandonment of terrains or livestock herding (Pini et al., 2021; Kolář et al., 2022). Botanical remains can provide information on agropastoral practices even without a precise taxonomical information. The analysis of density and shape of phytoliths found in coprolites allowed researchers to determine whether livestock was fed with wild or cultivated grasses, and even to identify to which grass subfamily the remains could belong (Dunseth et al., 2019). Information on introduction date and population trends could be highly relevant when working within the NPIH and AIAI frameworks, as native and invaded locations with a similar anthropogenic disturbance could be compared to verify whether the invasive species became more abundant in the latter region shortly after introduction, which would indicate pre-adaptation (e.g., European species increasing outside their native range - **NPIH prediction b**). Instead, by comparing samples in the same location but belonging to different periods, one could check whether there were alterations in community composition and relative species abundances after a target species introduction. Such information must be interpreted in light of the development of agropastoral practices in the region. For example, if an intensification of anthropogenic disturbance went alongside an increase in abundance of an invasive species, this could also provide an indication for pre-adaptation (**AIAI assumption c**, and **NPIH prediction c** in the case of agropastoralism as disturbance). Similarly, the community composition in neighboring sites with different intensities of disturbance could be compared to test whether European invasive species were able to spread even in the absence of disturbance (**NPIH prediction d**).

Depending on the research question of interest, after taxonomical identification, a variety of methods can be applied to extract further information from the remains. An avenue that holds a lot of promise for the study of invasive species’ evolution is the analysis of ancient DNA (aDNA). A single species’ genome can be characterized from micro- and macroremains using Next Generation Sequencing (**Figure 2B**) (Metzker, 2010). Some types of samples, such as sediment or coprolites, can contain several species’ microremains or fragments of macroremains that are too small to be identified and are therefore analyzed through metabarcoding or metagenomics, which can provide an overview of the past community’s taxonomic composition (**Figure 2B**) (Taberlet et al., 2012; Pedersen et al., 2016; Parducci et al., 2017). Excellent reviews have summarized the most recent aDNA analysis techniques, such as Hofreiter et al. (2015); Orlando et al. (2015), and Danielewski et al. (2023). Information on ancient species’ evolution can be gathered also from morphometric analyses of archaeobotanical macroremains, which, if applied to charred materials needs careful consideration due to the possible deformations as consequence of the charring process (Charles et al., 2015). The traditional approach is called “linear”, and consists of measurements of dimensions such as width, length, and thickness (**Figure 2B**). In the last decade, researchers developed geometric morphometrics, which converts shapes into quantitative variables using mathematical frameworks and provides a much more wide-ranging overview of a sample’s shape (Bonhomme et al., 2017), allowing for detailed comparisons with other samples (**Figure 2B**). For a comprehensive review on the topic see Noshita et al. (2022).

The implementation of aDNA analysis and morphometrics improve the level of identification and can further help to identify genes or traits that might have surged as adaptations to anthropogenic disturbance (**Figure 2C**). Many researchers support the idea that disturbed populations present traits such as short life spans, small and numerous seeds, and fast growth rates (Grime, 1977; Pierce et al., 2013; Salguero-Gómez et al., 2016). Due to the co-habitation with grazers, for example, plants might have evolved tolerance to trampling or strong resprouting abilities or resistance traits including mechanical defenses such as spikes or thorns. For some species, researchers have identified DNA regions that correlated to trampling tolerance (invasive wild rice *Oryza rufipogon* Griff. - Onishi et al., 2007) or compensatory growth after defoliation (invasive Italian ryegrass *Lolium perenne* L. - Lee et al., 2011). Grazing or mowing can also promote a decrease in seed size (Völler et al., 2013; Herben et al., 2018), and temporal variation in size could be easily verified through morphometrics. Through both aDNA and morphometric analysis it would be possible to verify whether past native populations did adapt to a newly introduced anthropogenic disturbance over time (**AIAI assumption a**), such as in the case of European plants exposed to agropastoralism after its introduction in the continent during the Neolithic (background assumption of **NPIH**). The application of such methods, for example, allowed researchers to establish in which historical period maize acquired and fixed some key domestication traits (unbranched plant architecture, storage protein synthesis, and starch production) in its native range of Mexico (Jaenicke-Després et al., 2003) and how it then regionally adapted to the environmental conditions while being transported northwards from Central America by early farmers (da Fonseca et al., 2015). Another study highlighted instead a decrease in length and a change in shape of lentil seeds (*Lens culinaris* Medik.) when comparing samples from the Early Iron Age (625–575 BCE) to those from the Middle Iberian period (400–200 BCE), possibly due to a change in irrigation practices (Tarongi et al., 2021). These methodologies would also allow for a comparison of traits and genes between pre-adapted invasive species and closely related species found in their invaded range (e.g., European species vs. North American species - **NPIH prediction c**) and for verifying whether this distinction was preserved over time or if it was lost due to adaptation of the local community or confounded by post-introduction evolution (**Figure 2C**).

Finally, some of the previous research on invasive species is based on materials from herbaria and focus on intercontinental human-mediated translocation, which can help identify introduction dates and genetic sources of invasive populations – a key step to test the hypotheses related to pre-adaptation and invasions (e.g., **AIAI assumption b**). For example, by comparing DNA from contemporary populations and 19^th^-century herbaria samples, researchers were able to determine that the European invasive species *Arabidopsis thaliana* (L.) Heynh. was introduced in the United States around 400 years ago (Exposito-Alonso et al., 2018). DNA from herbaria samples also shed light on the past evolution of two genetic clusters of *Ambrosia artemisiifolia* in its native range, one of which was strongly associated with agricultural disturbances and is the source for some invasive populations in France and Hungary (Martin et al., 2014). Nonetheless, the collection of dry plant material in herbaria dates back no further than the 16^th^ century (Baldini et al., 2022), which limits the temporal range of material that can be used to the last 500 years. However, the techniques implemented in these studies should be applicable also to older plant remains, which opens a world of exciting possibilities for ecologists working on wild plants’ evolution.

## 6 Concluding remarks

Pre-adaptation to anthropogenic disturbance is assumed to favor the aggressive spread of plant invaders, especially in agropastoral ecosystems. The proposed strategies to test the complex related hypotheses focus on contemporary populations, which does not allow us to fully extrapolate the past effect of anthropogenic disturbance as a selective force acting millennia ago. A collaboration between ecology and archaeobotany has the potential to uncover even more information about past adaptation processes and that is what we strongly promote here. Remains of wild species have been used in recent decades to extrapolate information on management practices but are rarely used in the context of evolutionary ecology. They often reach back several millennia and can thus provide valuable information far beyond the common scope of ecological experiments (~20 years in the past at most) by helping reconstruct past environmental conditions, species introductions, extinctions, and changes in the relative abundance of native and invasive species in relation to ecosystem changes. By applying methodologies from genomics and morphometrics to the same archaeobotanical samples, we can identify genes and traits that surged during the process of adaptation to anthropogenic disturbances such as agropastoralism and identify precisely at what point in time they appeared. The application of such approaches requires collaboration networks combining the skills of archaeobotanists, ecologists, geneticists and archaeologists (e.g., Filatova et al., 2021; Jesus et al., 2021; Salavert et al., 2022). Such projects should be designed for particular invasive species, following their evolution through the different environmental changes both in their native range and subsequently while establishing and expanding in the invaded range.

## Author contributions

GB: Conceptualization, Visualization, Writing – original draft, Investigation. KS: Writing – original draft, Conceptualization, Investigation, Methodology, Supervision, Validation. WK: Writing – review & editing, Funding acquisition. AE: Supervision, Writing – review & editing, Conceptualization, Funding acquisition, Resources, Validation, Methodology.
